# Isolated Otolith Dysfunction in Persistent Postural-Perceptual Dizziness

**DOI:** 10.3389/fneur.2022.872892

**Published:** 2022-04-11

**Authors:** Toshihisa Murofushi, Koji Nishimura, Masahito Tsubota

**Affiliations:** ^1^Department of Otolaryngology, Teikyo University School of Medicine, Mizonokuchi Hospital, Kawasaki, Japan; ^2^Department of Otolaryngology, Kanazawa Medical University, Himi Municipal Hospital, Himi, Japan

**Keywords:** PPPD, VEMP, vHIT, isolated otolith dysfunction, DHI, NPQ, idiopathic otolithic vertigo

## Abstract

The aims of this study were to investigate otolith dysfunction, especially isolated otolith dysfunction (with preserved semicircular canal function) in persistent postural-perceptual dizziness (PPPD) patients. Twenty-one patients who had been diagnosed with PPPD were enrolled in this study. The subjects filled out questionnaires [the Dizziness Handicap Inventory (DHI) and the Niigata PPPD Questionnaire (NPQ)] and underwent vestibular evoked myogenic potential (VEMP) tests, video head-impulse tests (vHIT), and stabilometry. Among the 21 subjects with PPPD, 9 showed isolated otolith dysfunction, 4 exhibited both otolith dysfunction and semicircular canal dysfunction, and 2 demonstrated isolated semicircular canal dysfunction. Six subjects exhibited normal VEMP and vHIT results. Concerning the subjects' questionnaire scores and stabilometric parameters, there were no significant differences among subgroups when the subjects were classified according to their VEMP and vHIT results while stabilometric parameters obtained in PPPD subjects were significantly increased than published data of healthy subjects. As precipitating conditions for PPPD, vestibular neuritis was the most frequent and the second most was idiopathic otolithic vertigo. In conclusion, the majority of PPPD patients had otolith dysfunction, and most of them showed isolated otolith dysfunction. Idiopathic otolithic vertigo can be a precipitating factor of PPPD. While otolith dysfunction may be associated with initiation of PPPD symptoms, PPPD symptoms are also considered to be associated with other dysfunctions of the sensory processing system.

## Introduction

Persistent postural-perceptual dizziness (PPPD) is characterized by chronic dizziness and/or non-spinning vertigo, mainly in the upright position during standing or walking (1). PPPD is typically preceded by acute vestibular disorders. As non-spinning vertigo can be caused by disorders of the otolith organs, which senses linear acceleration (2–5), PPPD may be associated with otolith dysfunction rather than semicircular canal dysfunction. There have been some reports about otolith dysfunction in PPPD, which were based on vestibular evoked myogenic potential (VEMP) testing (6, 7), a clinical test of otolith organ function (5, 8). However, they did not focus on isolated otolith dysfunction; i.e., abnormal otolith function with preserved semicircular canal function.

Herein, we studied peripheral vestibular dysfunction in patients that had been diagnosed with PPPD using VEMP testing and the video head impulse test (vHIT), which is a clinical test of the semicircular canal, which senses angular acceleration (9, 10).

## Materials and Methods

### Subjects

Twenty-one patients (6 males and 15 females) that had been diagnosed with PPPD according to the diagnostic criteria outlined by the Barany Society (1) were enrolled in this study. The ages of the subjects ranged from 26 to 81 (mean = 59.0). Subjects with external and/or middle ear problems were excluded.

For reference, stabilometric findings in PPPD patients were compared with 55–59-year-old healthy subjects. We used data published in Japan that were obtained with the same methods as were employed in this study (11). Specifically, we used data for healthy subjects aged 55–59 years (45 females and 24 males) because the mean age of subjects in this study was 59.0.

### Methods

The subjects underwent cervical and ocular vestibular evoked myogenic potential (cVEMP and oVEMP) testing, vHIT testing, and stabilometry (5, 8–10, 12, 13). They also filled out 2 questionnaires, the Dizziness Handicap Inventory (DHI) (14) and the Niigata PPPD Questionnaire (NPQ) (15).

#### VEMP Testing

##### cVEMP

Electromyographic (EMG) signals were recorded using surface electrodes placed on the upper half of each sternocleidomastoid muscle (SCM) (active), with a reference electrode placed on the lateral end of the upper sternum. While in the supine position, the subjects were asked to raise their heads to contract the SCM. The EMG signals were amplified and bandpass-filtered (20–2,000 Hz) using the Neuropack system (Nihon Kohden, Japan). Short tone bursts (500 Hz air-conducted, 125 dBSPL, rise/fall time: 1 ms, plateau time: 2 ms) were used for stimulation at a repetition rate of 5 Hz. The analyzed period was 100 ms long (20 ms before and 80 ms after the stimulus). The rectified EMG signals obtained during the pre-stimulation period were used to assess background muscle activity (16, 17).

The amplitude of p13-n23 (the first positive-negative deflection) and the latency of p13 were analyzed. The normalized amplitude (NA) was calculated as the p13-n23 amplitude divided by background muscle activity. Background muscle activity was calculated using rectified EMG signals obtained during the pre-stimulation period (−20 to 0 ms). Asymmetry ratios (AR) for the cVEMP were calculated as follows: AR = 100 × (NAl – NAs)/(NAl + NAs), where NAl represents the NA on the larger response side, and NAs represents the NA on the smaller response side. The upper limit of normal for the AR was set at 41.6 (16). The upper limit of normal for the p13 latency was set at 17.7 ms (p13) (16).

##### oVEMP

EMG signals were recorded using surface electrodes placed 1 cm below the center of each lower eyelid (active) and 2 cm below the active electrode (reference). During recordings, the subjects were instructed to maintain an upward gaze. Bone-conducted stimulation (500 Hz, rise/fall time: 1 ms, plateau time: 2 ms) was presented using a 4810 mini-shaker (Bruel & Kjaer, Denmark) placed on the Fz position at a repetition rate of 5 Hz. The peak driving voltage was adjusted to 8.0 V, which produced a peak force level of 128 dB (re: 1 μN). The signals were amplified and bandpass-filtered (20–2,000 Hz) using the Neuropack system. The raw amplitude of N1-P1 (the first negative–positive deflection) was analyzed. The upper limits of normal for the AR and N1 latency were set at 27.3 (AR) and 11.7 ms (N1 latency) (17).

#### vHIT

The Eye-See-Cam system (Interacoustics, Denmark) was used for the vHIT. The patients were subjected to passive high-acceleration, low-amplitude head rotations in the planes of the lateral, right anterior-left posterior, and left anterior-right posterior semicircular canals (9, 10, 18). Each subject was seated 1.5 m in front of a target and asked to keep watching it as their head was passively rotated by the examiner. Their eye and head movements were measured using video-oculography and inertial sensors. The vestibulo-ocular reflex (VOR) gains that occurred during the testing (eye velocity/head velocity) were measured using software. When a mean gain during the vHIT (eye velocity/head velocity) of <0.7 for the vertical canals or <0.8 for the lateral canals was detected, the relevant canal was regarded to be functioning abnormally (10).

#### Stabilometry

We used the Gravicorder GW-31 (Anima Co. Ltd., Japan) for the stabilometric recordings. It contains vertical force transducers, which can be used to measure instantaneous fluctuations in the center of pressure (COP). The stabilometric measurements were performed in both eyes-open and eyes-closed conditions for 60 s each. The positions of the subjects' feet and arms during the recordings were based on the standard Japanese methods (13). The subjects' feet were placed in the closed-parallel position, and their arms were extended laterally.

The outcome measures were the total length of sway of the COP over 60 s while the subjects had their eyes open or closed (Lo and Lc, respectively) and the enveloped area traced by the sway of the COP over 60 s while the subjects had their eyes open or closed (Ao and Ac, respectively).

#### Questionnaires

We used the DHI and NPQ for assessing subjective symptoms. The original DHI was developed by Jacobson and Newman (14) to assess handicaps in the daily lives of patients with balance problems. The NPQ was developed by Yagi et al. (15) as a tool for assessing the symptoms of PPPD. In this study, subjective symptoms were assessed based on the total score for each questionnaire.

#### Statistical Methods

For comparisons among the patients, the Kruskal-Wallis test was used. For comparisons between the patients in this study and the published data for healthy subjects, the *t*-test was used. *P*-values of <0.05 were regarded as statistically significant.

We obtained informed consent from each participant. This study was approved by the ethics committee of Teikyo University (TR20-078).

## Results

### Isolated Otolith Dysfunction

**Figure 1:**
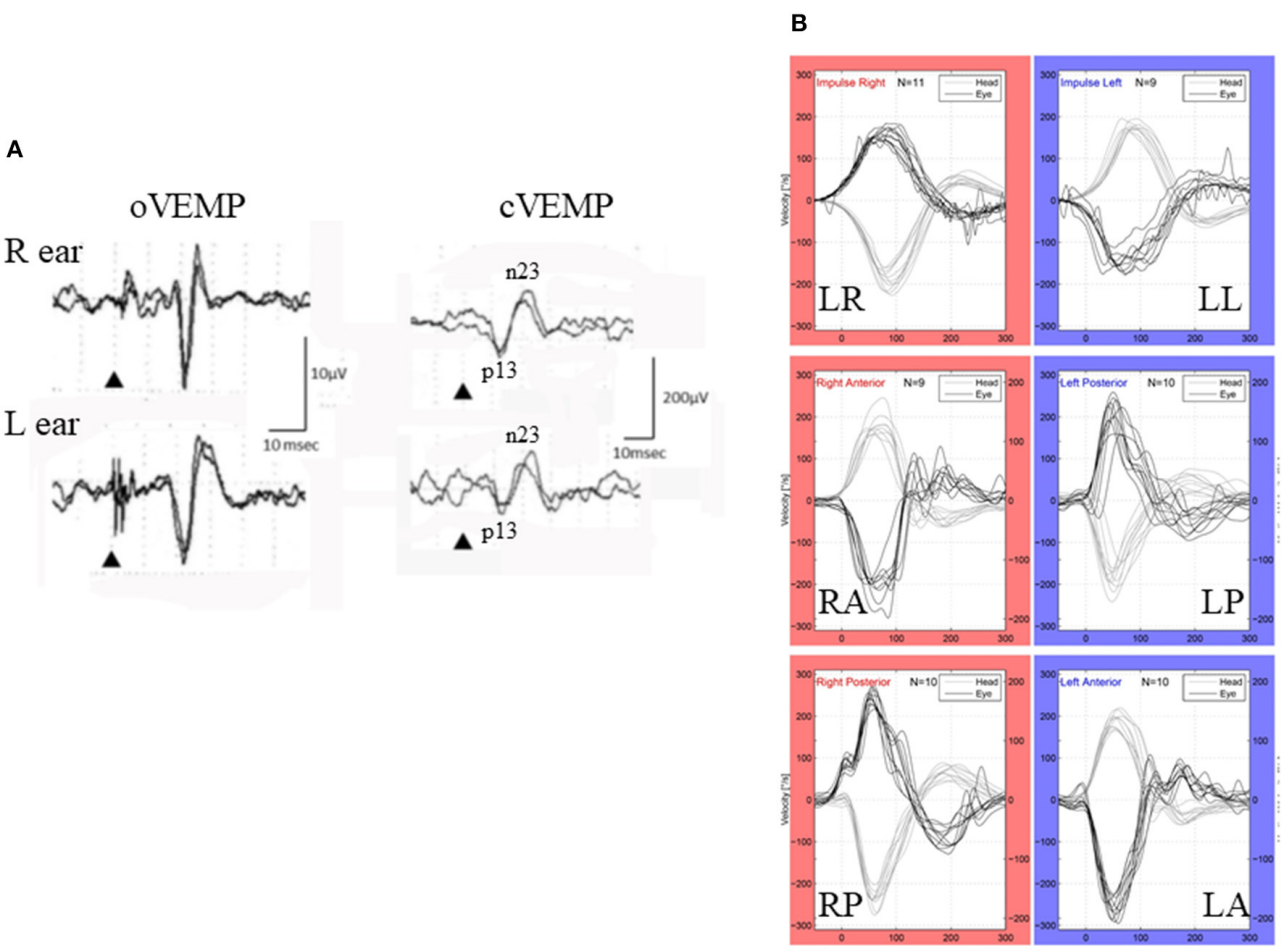
VEMP and vHIT (patient # 9, 25–29-year-old-male). **(A)** cVEMP and oVEMP. While he had normal cVEMP on both sides (right), he showed absence of oVEMP on both sides (left). Although he showed some positive-negative biphasic deflections with long latencies (>20 ms), they were not regarded as N1-P1 of the vestibular origin because of their reversed polarity and long latency. **(B)** vHIT. He showed normal responses to all the canals.

Among the 21 subjects, 9 (42.8%) showed isolated otolith dysfunction (Table 1; Figure 1). Four subjects demonstrated dysfunction of both the otolith organ and semicircular canal, 2 exhibited isolated semicircular canal dysfunction, and 6 showed normal peripheral vestibular end-organ function. Therefore, 13 subjects (61.9%) had otolith dysfunction, 6 (28.5%) had semicircular canal dysfunction, and 6 (28.5%) showed normal functioning of both the semicircular canal and otolith organ.

**Table 1:** Summary of patients with isolated otolith dysfunction.

**No**.	**Age**	**Sex**	**DHI**	**NPQ**	**cVEMP**	**oVEMP**
1	70–74	F	40	32	Unil. absent	Unil. absent
2	60–64	F	34	33	Normal	Unil. absent
3	25–29	F	46	37	Normal	Unil. decreased
4	70–74	F	42	42	Unil. decreased	Normal
5	80–84	M	78	36	Unil. absent	Unil. absent
6	50–54	F	64	64	Unil. absent	Normal
7	65–69	F	44	36	Unil. absent	Bil. absent
8	70–74	F	52	31	Normal	Unil. decreased
9	25–29	M	54	46	Normal	Bil. absent

Among the 9 subjects with isolated otolith dysfunction, 2 exhibited bilateral abnormal responses, while 7 exhibited unilateral abnormal responses. Three of the 9 subjects with isolated otolith dysfunction showed both abnormal cVEMP and oVEMP responses. In 4 subjects, normal cVEMP were seen, but oVEMP were absent. On the other hand, in 2 subjects cVEMP were abnormal, but normal oVEMP were observed.

### Precipitating Conditions for PPPD

Precipitating conditions for PPPD are summarized in Table 2. Vestibular neuritis (VN) was the most frequent as precipitating condition (*N* = 7, 33.3%). The second most was idiopathic otolithic vertigo (OV) (*N* = 4, 19.0%). The diagnosis of OV was done according to symptoms in diagnostic criteria proposed by Sue et al. (19).

**Table 2:** Precipitating conditions.

**Precipitating condition**	**Only O**	**Both O and C**	**Only C**	**Normal**	**Total**
VN	1	3	1	2	7
OV	3	0	0	1	4
VM	2	0	0	0	2
MD	0	0	0	1	1
SD	0	1	0	0	1
BPPV	1	0	0	0	1
Unknown	2	0	1	2	5
Total	9	4	2	6	21

*C, semicircular canal dysfunction; O, otolith dysfunction; VN, vestibular neuritis; OV, idiopathic otolithic vertigo; VM, vestibular migraine; MD, Meniere's disease; SD, sudden deafness; BPPV, benign paroxysmal positional vertigo*.

### Comparison of Isolated Otolith Dysfunction Patients With Other PPPD Subjects

Comparisons of the outcome measures are shown in Table 3. None of the examined parameters exhibited significant differences by the subgroups (*p* > 0.05 Kruskal-Wallis test). The PPPD patient group (*N* = 21) showed significantly larger values of the examined stabilometric parameters than the 55–59-year-old healthy group (*t*-test *p* < 0.01) (11).

**Table 3:** Summary of questionnaires and stabilometry.

**Subgroup**	***N***	**DHI**	**NPQ**	**Ao (cm^**2**^)**	**Ac (cm^**2**^)**	**Lo (cm)**	**Lc (cm)**
**(A) Comparison of the subgroups in PPPD**							
Both C and O	4	59.5 + 29.7	38.7 + 6.7	9.06 + 6.86	16.81 + 12.87	118.60 + 23.04	213.55 + 54.72
		66 (18–88)	38 (33–46)	8.56 (2.76–16.35)	16.08 (3.40–31.69)	116.72 (92.40–148.56)	215.17 (146.47–277.40)
Only O	9	50.4 + 13.5	39.6 + 10.3	6.92 + 5.65	11.54 + 8.76	148.52 + 89.57	222.35 + 134.62
		46 (34–78)	36 (31–64)	3.46 (1.45–16.74)	6.48 (1.64–23.27)	103.24 (59.76–288.33)	154.12 (88.09–439.76)
Only C	2	53.0 + 4.2	29.5 + 9.1	2.5 + 0.31	5.16 + 1.61	82.82 + 3.76	146.02 + 8.66
		53 (50–56)	29.5 (23–36)	2.5 (2.28–2.72)	5.16 (4.02–6.30)	80.82 (80.16–85.48)	146.02 (139.90–152.15)
Normal	6	57.0 + 15.9	43.8 + 15.2	7.00 + 9.71	10.53 + 15.65	97.81 + 47.82	125.37 + 69.17
		63 (28–72)	44 (22–61)	2.62 (1.21–21.54)	3.15 (1.88–33.96)	87.56 (52.17–163.98)	103.55 (71.33–223.07)
	***N***	**Ao (cm** ^**2**^ **)**	**Ac (cm** ^**2**^ **)**	**Lo (cm)**	**Lc (cm)**		
**(B) Comparison of PPPD patients with healthy 50–59-year-old population**							
PPPD	21	6.92 + 6.40	11.76 + 10.66	124.63 + 68.43	192.05 + 105.55		
HS F	45	3.57 + 1.74	4.66 + 2.25	78.93 + 15.82	137.93 + 28.91		
HS M	24	3.14 + 1.44	4.23 + 1.69	98.20 + 31.46	144.52 + 56.53		

*In (A) the upper row of each subgroup represents mean + SD and the lower row does median (range). HS F: 55–59-year-old female, HS M: 55–59-year-old male (11)*.

*PPPD, patients had significantly increased sway to all parameters shown to controls (p < 0.01)*.

## Discussion

PPPD is characterized by chronic dizziness and/or non-spinning vertigo, mainly in the upright position during standing or walking (1). PPPD is typically preceded by acute vestibular disorders. Therefore, PPPD seems to be associated with otolith dysfunction because the otolith organ senses linear acceleration and its dysfunction could lead to non-spinning vertigo. Adamec et al. (6) and Yagi et al. (7) studied otolith dysfunction using VEMP, they did not focus on isolated otolith dysfunction; i.e., abnormal otolith function with preserved semicircular canal function. Waterston et al. reported that 20 % of their PPPD patients showed isolated otolith abnormality (20). However, diagnoses of these patients with isolated otolith dysfunction were not shown. Although the ratio of isolated otolith dysfunction to the whole participants is different from ours (20 vs. 42%) and their report did not describe about the details of otolith dysfunction, their report is consistent with ours at the point that PPPD patients could have isolated otolith dysfunction.

Murofushi et al. reported episodic non-spinning vertigo with dysfunction of the otolith organ (2, 3). Murofushi proposed that episodic non-spinning vertigo with abnormal VEMP findings with preserved canal function could be called otolithic vertigo (5). The term “otolithic vertigo” or similar terms have been used by several investigators as well (21–24). Murofushi also proposed that otolithic vertigo which cannot be diagnosed with known vestibular diseases might be called “idiopathic otolithic vertigo” (OV) (5). The present study showed that idiopathic otolithic vertigo could be a precipitating factor of PPPD. Apart from acute or episodic forms of otolith dysfunction, otolith dysfunction may be a chronic condition. The chronic form of isolated otolith dysfunction may be closely associated with PPPD.

The present study showed that 13 of the 21 PPPD patients (61.9%) had otolith dysfunction. Among these 13 patients, 9 had isolated otolith dysfunction. Otolith dysfunction seems to be one of the causes of PPPD. In this study, OV was the second most condition precipitating for PPPD while the most was VN. Because both VN and OV should include acute otolith dysfunction, otolith dysfunction seems to contribute to the initiation of PPPD symptoms. However, there were no significant differences in stabilometric findings or questionnaire scores between isolated otolith dysfunction type and other subtypes of PPPD based on which vestibular end-organ was damaged at the time of PPPD. These findings suggest that the continuation of PPPD symptoms, such as floating dizziness during walking, might also require dysfunction of other areas of the sensory processing system than the peripheral vestibular system.

Yagi et al. reported that three clusters of PPPD patients were revealed: the visual dominant subtype, active-motion dominant subtype, and mixed subtype (7). The visual dominant subtype does not have a close relationship with damage to the peripheral vestibular system, while the active-motion dominant subtype may show associations with otolith dysfunction in a larger study. Failure to appropriately weight inputs from sensory modalities other than the vestibular system, such as the visual and/or somatosensory system, or excessive excitability of the vestibular system in response to fluctuations in inputs may play a role in PPPD.

The main limitation of this study is that the size is small. Large-sized study should be performed in the future. Furthermore, while this study showed that OV could be a precipitating factor of PPPD, there is still remained to be clarified how often OV will proceed to PPPD. In this cross-sectional study, symptoms and sway measured with stabilometry do not differ between subjects with isolated otolithic abnormalities and others. In order to determine if otolithic abnormalities affect the prognosis of patients with PPPD a follow up study is required. Thirdly, precipitating factors of PPPD might be biased by the department or institute where patients were seen. While Waterston reported that anxiety was the most as a precipitating factor and the second most was vestibular migraine (20), in the present study VN was the most and OV was the second most. Interdisciplinary and international survey might be required.

In conclusion, the majority of our PPPD patients had otolith dysfunction, and many of them showed isolated otolith dysfunction with preserved semicircular canal function. While otolith dysfunction may be associated with initiation of PPPD symptoms, persistence of symptoms might also require dysfunction of other areas of the sensory processing system than the peripheral vestibular system.

## Data Availability Statement

The original contributions presented in the study are included in the article/supplementary material, further inquiries can be directed to the corresponding authors.

## Ethics Statement

The studies involving human participants were reviewed and approved by Teikyo University Ethics Committee. The patients/participants provided their written informed consent to participate in this study.

## Author Contributions

TM wrote the manuscript. KN and MT reviewed and edited the manuscript. All authors contributed extensively to the work presented in this article and collected data.

## Funding

This study was supported by a Grant-in-Aid for Scientific Research (C) from the Japan Society for the Promotion of Science (19K09856).

## Conflict of Interest

The authors declare that the research was conducted in the absence of any commercial or financial relationships that could be construed as a potential conflict of interest.

## Publisher's Note

All claims expressed in this article are solely those of the authors and do not necessarily represent those of their affiliated organizations, or those of the publisher, the editors and the reviewers. Any product that may be evaluated in this article, or claim that may be made by its manufacturer, is not guaranteed or endorsed by the publisher.
